# Primary antibody response after influenza virus infection is first dominated by low-mutated HA-stem antibodies followed by higher-mutated HA-head antibodies

**DOI:** 10.3389/fimmu.2022.1026951

**Published:** 2022-11-03

**Authors:** Aafke Aartse, Daniella Mortier, Petra Mooij, Sam Hofman, Marlies M. van Haaren, Martin Corcoran, Gunilla B. Karlsson Hedestam, Dirk Eggink, Mathieu Claireaux, Willy M. J. M. Bogers, Marit J. van Gils, Gerrit Koopman

**Affiliations:** ^1^ Department of Virology, Biomedical Primate Research Centre, Rijswijk, Netherlands; ^2^ Laboratory of Experimental Virology, Department of Medical Microbiology and Infection Prevention, University of Amsterdam, Amsterdam, Netherlands; ^3^ Department of Parasitology, Biomedical Primate Research Centre, Rijswijk, Netherlands; ^4^ Amsterdam Institute for Infection and Immunity, Infectious Diseases, Amsterdam, Netherlands; ^5^ Department of Microbiology, Tumor and Cell Biology, Karolinska Institutet (KI), Stockholm, Sweden

**Keywords:** influenza A virus, hemagglutinin, HA-stem, HA-head, primary response, antibody response

## Abstract

Several studies have shown that the first encounter with influenza virus shapes the immune response to future infections or vaccinations. However, a detailed analysis of the primary antibody response is lacking as this is difficult to study in humans. It is therefore not known what the frequency and dynamics of the strain-specific hemagglutinin (HA) head- and stem-directed antibody responses are directly after primary influenza virus infection. Here, sera of twelve H1N1_pdm2009_ influenza virus-infected cynomolgus macaques were evaluated for HA-head and HA-stem domain antibody responses. We observed an early induction of HA-stem antibody responses, which was already decreased by day 56. In contrast, responses against the HA-head domain were low early after infection and increased at later timepoint. The HA-specific B cell repertoires in each animal showed diverse VH-gene usage with preferred VH-gene and JH-gene family usage for HA-head or HA-stem B cells but a highly diverse allelic variation within the VH-usage. HA-head B cells had shorter CDRH3s and higher VH-gene somatic hyper mutation levels relative to HA-stem B cells. In conclusion, our data suggest that HA-stem antibodies are the first to react to the infection while HA-head antibodies show a delayed response, but a greater propensity to enter the germinal center and undergo affinity maturation.

## Introduction

The seasonal influenza virus vaccines are suboptimal as they do not elicit broadly neutralizing antibodies that protect against potentially newly arising influenza virus strains. Part of the problem may be that previously encountered influenza virus infections shape the immune response to subsequent exposures or vaccinations (1–3), which makes it challenging to predict or improve vaccine efficacy. A better understanding on how antibody responses are formed after a primary influenza virus infection is therefore needed but challenging to investigate in humans because of their complex influenza virus exposure history starting at a young age. It is known that the antibody responses towards the stem domain of the glycoprotein hemagglutinin (HA) are subdominant in adults (4, 5) in comparison to the immunogenic head domain (5, 6). In general, head antibodies are potent but virus strain specific and stem antibodies are broadly reactive but less potent. Typically, the HA-stem and HA-head responses in humans reflect the combination of many exposures to different influenza virus strain infections and vaccination history. In animal studies it was shown that HA-stem responses can be induced after primary infection with the seasonal PR8/34 H1N1 strain (7). In addition, in humans an increase in HA-stem domain responses was demonstrated after exposure to heterologous HA either by vaccination or infection (8, 9). However, vaccination may also lead to preferential induction of HA-head responses (1, 10). A naive animal model, such as cynomolgus macaques (11–14), can be used to investigate the primary influenza virus antibody response towards the HA-stem and HA-head domains in detail, because human individuals experience multiple influenza virus infections already at young age.

Reliable tools are needed to study HA-specific B cells and serum antibodies, subdivided in HA-trimer, -head, and -stem. The recombinant ectodomain of HA-trimer and trimeric HA-stem proteins have been used to study HA-stem specific and non-stem responses (4, 15, 16). Recently, a monomeric HA-head protein was included to provide further insight in HA-specific antibody responses and non-stem, non-head responses were identified, designated as HA-trimer-only responses (16). The HA-proteins, including monomeric head- and trimeric stem-proteins, can be used to study serological responses using assays such as enzyme-linked immuno sorbent assay (ELISA) as well studying HA-specific B cells by flow cytometry. In-depth characterization of antigen-specific B cell responses through B cell receptor (BCR) sequence analysis requires an appropriate immunoglobulin germline gene reference database. For cynomolgus macaques, an extensive VH germline gene database generated with the IgDiscover tool was recently described (17, 18), highlighting a high allelic variation between animals. Thus, with the development of IgDiscover, it has become possible to create individualized germline databases for animals of specific interest for precise analysis of germline gene usage and somatic hypermutation (SHM) levels (18, 19). Together, these techniques allow comprehensive analyses of serological responses, antigen-specific B cell frequencies, and BCR sequences of HA-responses at an individual level.

In this study, the HA-trimer, HA-head, and HA-stem specific antibody responses after primary H1N1_pdm2009_ influenza virus infection were evaluated in twelve cynomolgus macaques (20) by ELISA and flow cytometry using the HA-proteins as described before (16). HA-trimer specific BCRs were characterized in depth for six animals using single B cell sorting followed by BCR sequencing. Combining the results of the specific antibody and B cell responses with the in-depth analysis of HA-specific BCRs revealed that HA-stem antibodies form the first antibody response following an acute influenza virus infection. At a later timepoint, HA-head-directed B cells and antibodies dominate, which showed higher SHM-levels compared to the HA-stem antibodies.

## Materials and methods

### Study setup and samples

The study was performed using serum and peripheral blood mononuclear cells (PBMCs) obtained from twelve cynomolgus macaques that had been infected *via* intrabronchial route with influenza virus strain A/Mexico/InDRE4487/2009 (Mex4487; H1N1_pdm2009_), using either a standard dose of 4x10^6^ or a higher dose of 4x10^8^ TCID_50_ as described previously (20). The study was approved by the Institutional Animal Care and Use committee of Biomedical Primate Research Centre (dierexperimentencommissie, DEC-BPRC; DEC#686). Serum samples were collected prior to infection (day 0) and at days 14- and 56-days post-infection. PBMCs were collected at day 56 post-infection only. From animal A02 and A06 also pre-infection PBMCs were available. PBMCs were isolated by density gradient centrifugation separation using LeucoSep tubes (Greiner Bio-One) and freshly prepared Percoll density gradient (1.078 g/mL) medium (Cytiva, Uppsala, Sweden) diluted with 10X Dulbecco’s Phosphate Buffered Saline (DPBS, Gibco) and Water for Injection (WFI) for cell culture (Gibco). The cells were cryopreserved in 10% dimethyl sulfoxide (DMSO, Merck) in Fetal Bovine Serum (FBS, Greiner Bio-One) until further analysis. Untreated blood was left to clot for at least 30 min at room temperature. After centrifugation for 10 min at 2,200 rpm, serum was collected and stored at -80°C until further analysis.

### Recombinant protein design, production, and purification

The recombinant A/Netherlands/602/2009 HA-trimer, HA-monomeric head, and HA-trimeric stem proteins were designed (21), produced, and purified as described previously (16). Both HA-trimer and HA-head contain the Y98F mutation (22, 23). Briefly, all proteins were expressed in a mammalian cell line HEK293F (Invitrogen, cat no. R79007), and purified with nickel-agarose (Ni-NTA) beads columns (Qiagen, Venlo, the Netherlands), according to manufacturer’s instructions (Qiagen, the QIAexpressionist 06/2003), and buffer exchanged to Phosphate Buffered Saline (PBS, Gibco). Size exclusion chromatography (SuperDex 200 10/300 GL increase column) was used to collect the correct sized proteins, followed by examining the collected and pooled fractions by an SDS-PAGE and ELISA with well characterized mAbs to confirm the conformation of each protein. A fraction of the purified proteins was biotinylated *in vitro* with the BirA kit (GeneCopoeia, tebu-bio, Heerhugowaard, the Netherlands) as described previously (16). The biotinylated and non-biotinylated proteins were stored in PBS at -80°C.

### Enzyme-Linked Immuno Sorbent Assay

An Enzyme-Linked Immuno Sorbent Assay (ELISA) was performed to analyze antibody responses in serum. Ni-NTA plates (HisSorb, Qiagen) were coated with 1 µg/mL recombinant proteins (final concentration) overnight at 4°C, then washed with PBS (Gibco) followed by residual blocking step of 1 h with 2% nonfat dried milk powder (PanReac Applichem) in PBS (w/v). After washing with PBS, the sera to be analyzed were added in a three-fold serial dilution (starting at 1:100 diluted serum or 3 µg/mL control mAbs) in 2% milk/PBS, and plates were incubated for 90 min at room temperature, followed by a PBS wash. The secondary antibody (goat anti-human IgG, 1 mg/mL, KPL) labelled with horseradish peroxidase (HRP) was diluted 1:10,000 in 2% milk/PBS and incubated for 45 min. Next, a washing step was performed with 0.05% Tween20 (Merck) in PBS, the substrate (Chrom TMB, DIAsource ImmunoAssays SA, Belgium) was added, and color development was stopped with stop solution (Stop Solution, DIAsource ImmunoAssays SA, Belgium). The readout was done using a BioTek ELX800 microplate reader (BioTek Instruments, Vermont, U.S.A.) and the Optical Density (OD) was measured at 450 nm. Area under the curve (AUC) was measured from the linear regression line obtained from log transformed dilution range and OD_450nm_ in duplicate in Prism 9, statistical differences between timepoints of each protein coating were determined by using the Wilcoxon test.

For the competition ELISA, Ni-NTA plates were coated with HA-stem protein (1 µg/mL) overnight at 4°C, then washed with PBS followed by residual blocking step of 1 h with 2% milk/PBS. After washing with PBS, two representative sera were added in 1:50 dilution or mAb C179 (Takara) in 20 µg/mL. After 30-60 min incubation, mAb C179 was added to the sera (final dilution: 1:100, C179: EC_50_) and sera to the mAb C179 (final dilution: EC_50_, C179: 10 µg/mL), and plates were incubated for 90 min followed by a PBS wash. The secondary antibody (goat anti-mouse, 1 mg/ml, Sigma) labelled with HRP was diluted 1:1,000 in 2% milk/PBS and incubated for 45 min followed by the washing steps, developing, am readout as described above. Percentage of C179 binding was calculated relative to the maximum binding of C179.

### Single B cell sorting by flow cytometry

Single HA-trimer specific B cells from pre- and post-infection samples were sorted as described previously (16). Briefly, biotinylated HA-trimer, trimeric HA-stem, and monomeric HA-head proteins were conjugated in an 8:1 ratio (w/w) to streptavidin-conjugates BV605 (0.1 mg/mL, BioLegend), BV421 (0.1 mg/mL, BioLegend), or AF647 (0.5 mg/mL, BioLegend) respectively, resulting in fluorescent probes. Conjugation was performed in subdued light at 4°C for 90 min. PBMCs were then thawed and stained with the following surface markers: IgG-BB515 (G18-145, BD Bioscience), IgM-PE (G20-127, BD Bioscience), CD3-PerCP (SP34-2, BD Bioscience), CD14-ECD (RM052, Beckman Coulter), CD20-APC-H7 (L27, BD Bioscience), live/dead marker Aqua (Invitrogen), followed by adding the conjugated probes. The cells were incubated at 4°C for 30-60 min and washed with 0.5% FBS/PBS.

Live, CD3-, CD14-, CD20+, and HA-trimer+ single cells were sorted on a FACS ARIAIIIu flowcytometer into an empty 96-wells plate or containing 20 μl RT-lysis buffer consisting of 20 U RNAse inhibitor (Invitrogen), first strand superscript III buffer (Invitrogen), and 1.25 μl of 0.1 M DTT (Invitrogen). The plates were stored at -80°C before performing the reverse transcription as described before (16).

To accurately compare the frequency of monomeric HA-head versus trimeric HA-stem probe binding HA-trimer specific B cells, additional staining experiments were performed for the post-infection samples. The streptavidin-conjugates BV421 in a 4:1 ratio (w/w) and APC (0.2 mg/mL, BioLegend) in an 8:1 ratio (w/w) were used for the monomeric HA-head and trimeric HA-stem probe and vice-versa. The frequency of HA-head and HA-stem was evaluated for BV421-conjugates. The frequency of HA-trimer-only, HA-head+stem, IgG, and IgM B cells was the average of these two staining strategies. Data analysis was done with FlowJo v10.8.1. Statistical differences between binding domains were determined by using the Wilcoxon test.

### Immunoglobulin gene sequencing

cDNA was generated from the single cell sorted B cells as described before (16). Cells were sorted into 20 μl lysis buffer (stored at -80°C) or this was added afterwards (first stored at -80°C, followed by adding lysis buffer, and cells were lysed for 15-30 minutes on ice), then adding 6 μl of a mix of SuperScriptIII (Invitrogen), 200 ng hexamer primers (Invitrogen), and 6 mM dNTPs (Invitrogen) followed by RT-PCR program (16). Subsequently, the IgG and IgM variable regions of the heavy chain (HC) and light chains (LC, kappa or lambda) of the B cell receptor in three subsequent PCR reactions as described previously (24, 25). In short, the first HC PCR reaction was performed with 2 μl input cDNA and the following PCR reaction mix: 0.1 μl forward and reverse multiplexed primer mix (equimolar diluted), 0.3 μl dNTPs (10 mM), 0.075 μl HotStarTaq plus polymerase (5 U/μl, Qiagen), and 10x PCR buffer in a final reaction volume of 14.5 μl. PCR program used: 95°C 5 min; 50 cycles of 94°C, 30 s; 55°C, 30 s; 72°C for 1 min; and a final step of 72°C for 10 min. The second HC PCR reaction was performed with 2 μl DNA from first HC PCR and the following PCR reaction mix: 0.2 μl forward and reverse multiplex primer mix (equimolar diluted), 0.1 μl MyTaq DNA polymerase (5 U/μl, Meridian Bioscience), and 5x MyTaq reaction buffer in a final reaction volume of 20 μl for 1 min at 95°C, 50 cycles of 95°C for 15 s; 55°C for 15 s; 72°C for 45 s, and a final step of 72°C for 10 min. The last HC PCR was performed with primers containing an overlapping region designed for the ligation into the corresponding expression vector. This final nested HC PCR reaction was performed with 1 μl input of the second HC PCR reaction and 0.1 μl Phusion HiFi (2 U/μl, New England Biolabs) or HotStarTaq HiFidelity (2,5 U/μl, Qiagen), 5x Phusion GC or HotStarTaq HiFidelity buffer, 0.3 μl dNTPs (10 mM) only needed for Phusion HiFi, 0.05 μl forward and reverse multiplex primer mix (equimolar diluted) for Phusion HiFi or 0.2 μl forward and reverse multiplex primer mix for HotStarTaq HiFidelity in a total volume of 15 μl or 14.5 μl for HotStarTaq HiFidelity. For Phusion HiFi the following PCR program was used; 98°C, 30 s; then 35 cycles of 98°C, 5 s; 58°C, 15 s; 72°C, for 20 s; final elongation 10 min at 72°C. For HotStarTaq HiFidelity the following PCR program was used; 95°C, 5 min; then 50 cycles of 94°C, 15 s; 58°C, 1 min; 72°C, for 1 min; final elongation 10 min at 72°C.

The first and second LC PCR was performed with MyTaq DNA polymerase (5 U/μl, Meridian Bioscience) as described for heavy chain PCR2, with forward and reverse multiplex primer mix specific for kappa or lambda chain (equimolar diluted) and 2 μl input of either cDNA or PCR 1. Annealing temperatures of the first PCR were adapted to 52°C for kappa chain or 56°C for lambda chain. And the annealing temperatures of the second LC PCR were adapted to 53°C for kappa chain or 55°C for lambda chain. The final nested PCR was performed with 2 μl DNA of the second PCR reaction and the following reaction mix: 0.075 μl HotStarTaq plus polymerase (5 U/μl, Qiagen), 10x PCR buffer, 0.3 μl dNTPs (10 mM), 0.1 μl forward and reverse multiplexed primer mix specific for kappa or lambda chain (equimolar diluted), in a total volume of 14.5 μl. The used PCR program was: 95°C, 5 min; then 25 cycles of 94°C, 30 s; 58°C for both kappa or lambda chain for 30 s; 72°C for 1 min; final elongation 10 min at 72°C. The product of the last PCRs was scored positive or negative on a 1% agarose gel.

The ligation of the V(DJ-PCR product into a human IgG PSF-CMV-human vector (Merck) containing IgG-secretion signal peptide (introduced by Q5-site-directed-mutagenesis according to manufacturer’s protocol (New England Biolabs)) was done with Gibson Assembly mix (New England Biolabs). In short, the expression vectors were digested with restriction enzymes 10 U AgeI and 10 U BseRI or 10 U BsrBI (all New England Biolabs) per mg of vector. Digested expression vectors were extracted from 1% agarose gel using the Zymoclean Gel DNA Recovery Kit (Zymo Research). Ligation of the PCR products with the vectors was performed using the Gibson assembly method. Gibson assembly reaction was performed with 1 μl PCR product of the final reaction, 1 μl digested HC or LC vector (30 ng/μl), and 2 μl 2X Gibson Assembly Master Mix (New England Biolabs) and incubated at 50°C for 60 min. The plasmid DNA was then transformed into α-select *Escherichia coli* (*E. coli*) (New England Biolabs) and the insert of the recombinant V**(D**J-region was verified by Sanger sequencing in duplicates or triplicates (performed by Macrogen B.V., Amsterdam, the Netherlands).

The correct heavy or light chain sequence were first checked for productivity by using the default IMGT database (program version: 3.5.30 (28 March 2022), reference directory release: 202214-2 (05 April 2022)) (26, 27) for cynomolgus macaques for the heavy chain or rhesus macaques for the light chain. Productive sequences were then selected based on the most conserved sequence on amino acid and/or nucleotide level between duplicates or triplicates per monoclonal antibody (mAb) sequence for each animal to exclude potential PCR-errors (28). In addition, a sequence was excluded from analysis when a difference in the duplicates was observed in nucleotides of the highly variable region of the CDRH3. A sequence was included when only one sequence was available per heavy or light chain, with the unknown potential PCR-errors. Heavy chains were sequenced, and a selection was done based on binding domain, CDRH3-length, and isotype prior amplifying the corresponding light chains from that sorted B cell. Relative expression of the HA-specific BCRs was calculated per animal. Data handling and visualization was carried out with R version 1.4.1717.

### HEK293T monoclonal antibody screening

Screening of the isolated mAbs was done by using the mammalian expression system Human Embryonic Kidney cells (HEK293T) as described previously (16, 29). In short, HEK293T cells were cultured at 37°C with 5% CO_2_ in flasks with 1X DMEM + Glutamax (4.5 g/L D-glucose) (Gibco) supplemented with 10% FBS, streptomycin (100 μg/mL) and penicillin (100 U/mL) (both Gibco). HEK293T cells were seeded one day prior infection in 24-wells plate. The transfection was done with PEI (0.5 mg/mL, Merck) and heavy and light chain plasmid pair in a 4:1:1 ratio in a total volume of 50 μL OptiMEM (Gibco). The supernatant was harvested after two days and screened on IgG production and HA-trimer, monomeric head, and trimeric stem binding by ELISA. All HA-trimer binding Abs were confirmed in a second transfection scored as positive when the OD_450nm_ was two times above background signal in both experiments.

### Creating reference VDJ-sequences with IgDiscover

VDJ-germline sequences for each animal were defined as described previously (17, 18, 30). Briefly, IgM libraries were made with both leader- and UTR-specific primer sets. The libraries were sequenced with Illumina MiSeq at Leiden Genome Technology Centre (LGTC, Leiden, the Netherlands). IgDiscover version 0.15.1 was run for each leader- and UTR-library with an initial cynomolgus macaque VDJ-database of KIMDB v1.1 (17, 31, 32) with default settings except for 3’MTPX barcode (21 NT), pre-defined CDRH3-length (-160, -126), and renamed.

The usage levels of the IgM germline alleles were obtained by using the expressed.tab in the final folder after manual curation. Relative usage expression was calculated per animal and primer set, followed by adding up the allele expression per gene. Then, relative gene expression from both primer sets were compared and the highest relative expression per gene was used.

The final obtained leader and UTR VDJ-databases were combined for each animal and used in IgBLAST for evaluating the VDJ-usage and SHM of the HA-specific BCR sequences for each animal. SHM-levels were determined by using primer-trimmed HA-specific BCR sequences. Twenty or 22 nucleotides were trimmed from respectively 5’ and 3’ end of the HA-specific VDJ-sequences. The IMGT CDRH3 annotation was used for the CDRH3-length, meaning excluding the conserved cystine **(C)** and tryptophan (W) located 3’ and 5’ of the CDRH3. Distribution of the VH- and JH-gene families were calculated from total HA-trimer BCR sequences by counting number of sequences per gene-family. Significant differences of CDRH3-lengths or SHM-percentages between defined groups were calculated by a Mann-Whitney test. Data handling and visualization was caried out with R version 1.4.1717. Low numbers of HA-specific clonal families were found (<10) and were included in the analysis.

## Results

### Antibody responses against the stem and head domains of HA display different kinetics

**Figure 1:**
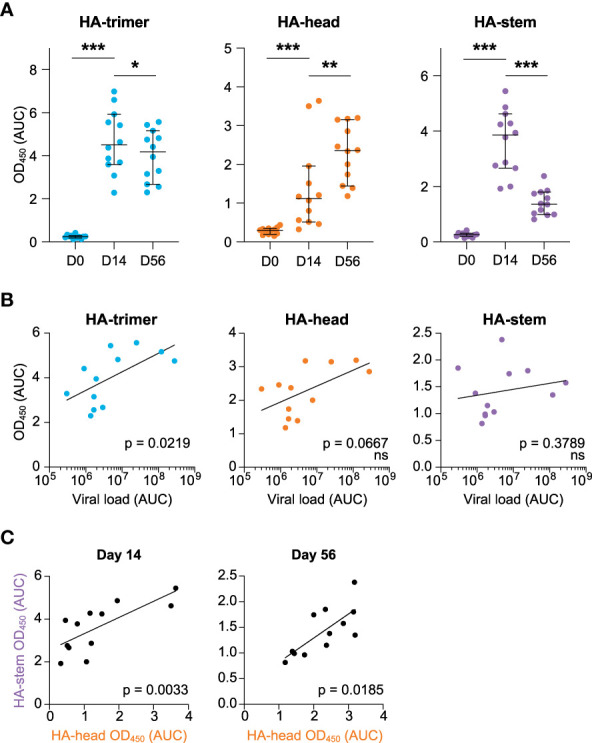
HA-antibody responses to H1N1_pdm2009_ at day 0, 14, and 56 post-infection **(A)** Antibody response measured in serum in time against the HA-trimer (blue), HA-head (orange), and HA-stem (purple) domains, calculated as area under curve (AUC), median and 95% CI are depicted **(B)** Correlation between cumulative viral load in tracheal swabs (AUC) and antibody responses against HA-trimer (blue), HA-head (orange), and HA-stem (purple) domain measured at day 56. **(C)** Correlation between HA-head and HA-stem antibody responses at day 14 (left) and day 56 (right). Statistical differences between two groups were determined using the Wilcoxon test (p values: *p < 0.05; **p < 0.01; ***p < 0.001) and correlations were determined using a Spearman correlation test (ns, not significant).

While it is assumed that the first encounter with influenza virus has a large impact on the immune response to subsequent exposures, such primary responses have not been well studied. To address this question, serum and PBMC samples from twelve cynomolgus macaques infected with an H1N1_pdm2009_ strain of influenza virus (20) were analyzed in detail. Serum antibody binding to HA-trimer, monomeric head domain, and trimeric stem domain were evaluated at different time points following acute infection: pre-infection (day 0), two weeks post-infection (day 14), and eight weeks post-infection (day 56) (**Figure 1** and **Figure S1**). Reactivity to HA-trimer provides insights in the total level of HA-specific antibodies, while reactivity to either the monomeric head domain or trimeric stem domain show reactivity to two distinct domains representing the two major classes of protective antibodies. The antibody responses towards the HA-trimer were rapidly induced and then decreased slightly from day 14 to day 56 (**Figure 1A**, left). At the same time-interval, the HA-head specific antibody response significantly increased (**Figure 1A**, middle) while the HA-stem specific antibody response after an initial increase at day 14, strongly decreased (**Figure 1A**, right). Specificity of binding to HA-stem was confirmed in two representative sera by competition ELISA using the anti-stem C179 mAb (**Figure S2**).

Interestingly, the viral load [as published (20)] was positively correlated with the overall HA-response at day 56 (**Figure 1B**, left), while no correlations were observed between HA-head domain or HA-stem domain responses at day 14 or day 56 and viral load (**Figure 1B**, right and **Figure S3**). This confirms that a higher virus production leads to higher antibody responses to the whole HA-glycoprotein, as noted before (20).

Nonetheless, there was a positive correlation between HA-head and HA-stem antibody responses both at day 14 and day 56 (**Figure 1C**). Hence animals that develop a strong response to HA-head also target the HA-stem domain.

### Primary infection with H1N1_pdm2009_-virus induces class switching of HA-specific B cells for both head and stem domains

**Figure 2:**
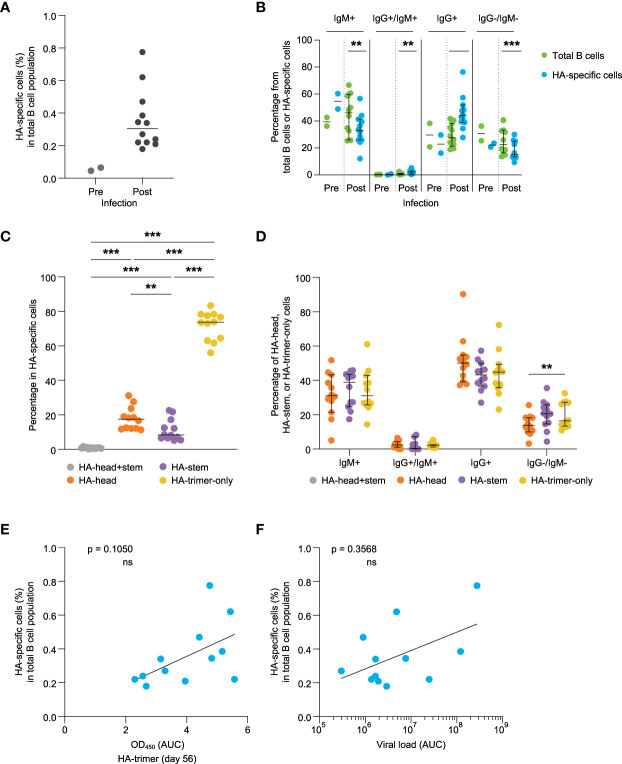
Evaluation of HA-specific B cell responses at day 56 post-infection **(A)** Percentage of the HA-specific B cells of pre- (green) and post-infection (blue) samples. **(B)** Comparison of the percentage of IgM+, IgG+/IgM+, IgG+, and IgG-/IgM- B cells detected in the total B cell pool (green) and HA-specific B cells (blue). **(C)** Frequency of HA-head+stem (grey), HA-head (orange), HA-stem (purple), and HA-trimer-only (yellow) binding B cells within the total HA-specific binding B cells. **(D)** Percentage of IgM+, IgG+/IgM+, IgG+, and IgG-/IgM- B cells observed within the HA-head+stem (grey), HA-head (orange), HA-stem (purple), and HA-trimer-only (yellow) populations of HA-specific cells. **(E)** Correlation between HA-specific antibody response (AUC) and frequency of HA-specific B cells measured at day 56. **(F)** Correlation between cumulative viral load in tracheal swabs (AUC) and frequency of HA-specific B cells at day 56. Statistical differences between two groups were determined using the Wilcoxon test (p values: **p < 0.01; ***p < 0.001), bars represent medians with 95% CI, and correlations were determined using a Spearman correlation test (ns, not significant).

Next, the induction of HA-specific B cell responses was investigated, using PBMCs collected at day 56 post-infection. Hence, the memory B cell response was studied which may deviate in some respect from the antibody response, which would rather represent an early post-infection plasmablast response. Using flow cytometry and HA-probes of HA-trimer, monomeric HA-head, and trimeric HA-stem (16) allowed for a detailed characterization of HA-specific B cell responses (**Figure S4**). Twelve animals’ post-infection PBMCs were analyzed and for two animals matched pre-infection samples were available and analyzed. The percentage of HA-specific cells in total B cells post-infection varied from 0.2% to 0.8%, while the two pre-infection samples were below 0.1% (**Figure 2A**). The isotype comparison of the total B cell pool (green) with the isotype distribution of the HA-specific B cells (blue) post-infection, showed that IgM+ cells were significantly decreased in the HA-specific cells while IgG+ cells were enriched in these cells compared to the total B cell pool (**Figure 2B**). This shows that HA-specific cells can undergo class switching after a primary influenza virus infection.

Most of the HA-specific B cells did not bind to either the head or stem and belonged to the HA-trimer-only subset as previously described for humans (16). A slightly higher percentage of B cells was observed to stain positive for the HA-head domain relative to the HA-stem domain at day 56 post-infection (**Figure 2C**). The isotype distribution of these subpopulations was not statistically significant different within IgG+ and/or IgM+ B cells (**Figure 2D**).

The percentage of HA-specific binding B cells did show a trend but did not reach significant correlation with HA-binding serum antibody levels at day 56 or day 14 (**Figure 2E** and **Figure S5**). Also, the HA-head binding antibody levels in serum did not correlate with the percentages of the HA-head binding B cells (**Figure S6**, two upper figures). However, for the HA-stem responses there was a significant correlation between HA-stem serum antibody levels and HA-stem specific B cell percentages at day 56 (**Figure S6**, lower right figure). The relatively late development of antibody responses against the HA-head domain might imply that they have not yet reached steady state levels and follow a slightly different time-path of development compared to their B cell response. The percentage of HA-trimer, HA-head or HA-stem binding B cells also did not correlate with the viral load (**Figure 2F** and **Figure S7**), further implying potential differences in development between the plasma cell and memory B cell response.

### Monoclonal antibodies confirm the observed binding specificities of the sorted HA-binding B cells

**Figure 3:**
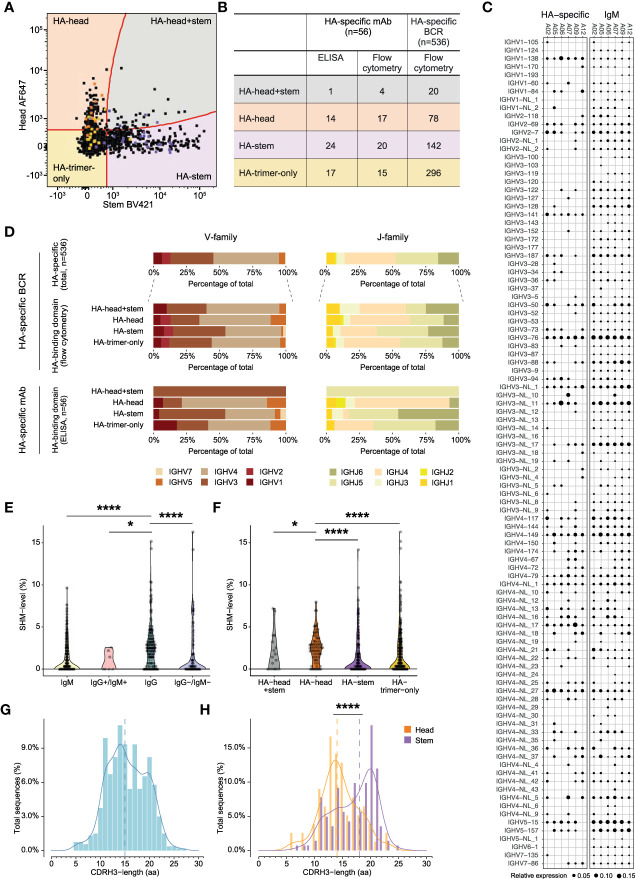
Characteristics of HA-specific BCR repertoire and monoclonal antibodies **(A)** Overlay flowcytometry plot of index-sorted HA-specific B cells of six animals. Original B cells of isolated monoclonal antibodies are highlighted according to ELISA binding pattern, in orange HA-head, in yellow HA-trimer-only, in purple HA-stem, and in grey HA-head+stem. **(B)** Table presenting the binding domain of HA-specific mAb in ELISA and flow cytometry and the binding domain HA-specific BCRs in flow cytometry. **(C)** BCR repertoire of IgM germline and HA-specific VH-gene usage. Size of the dots represent relative gene expression per animal. **(D)** Bar plots representing VH-gene (left) or JH-gene (right) family usage of HA-specific total BCRs, HA-head+stem, HA-head, HA-stem, and HA-trimer-only specific BCRs (upper panel) or isolated monoclonal antibodies (lower panel). **(E)** Violin plot showing VH-gene SHM-level of pre- (green) and post- infection HA-specific BCRs. Post-infection BCRs are shown in IgM+, IgM+/IgG+, IgG+, and IgM-/IgG- isotypes. **(F)** Violin plot showing VH-gene SHM-level of HA-head+stem (grey), HA-head (orange), HA-stem (purple), and HA-trimer-only (blue) specific BCRs. **(G)** Density histogram showing CDRH3-length in amino acids (aa) of total HA-specific BCRs. Dotted line represents the median CDRH3-length. **(H)** Density histogram showing CDRH3-length distribution of HA-head (orange) and HA-stem (purple) BCRs. Dotted lines represent corresponding medians. Statistical differences between two groups were determined using the Mann-Whitney test (p values: *p < 0.05; ****p < 0.0001).

Single HA-specific B cells were sorted from six animals (**Table S1**), and heavy and light chains of the B cell receptor (BCR) were amplified to verify the binding-specificity of these cells. The heavy and light chains were cloned into IgG expression vectors and subsequently produced as monoclonal antibodies (mAbs). Transfection supernatants harboring the mAbs (94.9% of total transfected mAbs) were tested in ELISA for binding to HA-trimer, HA-head, and HA-stem. Of these mAbs, 33.5% were found to bind in ELISA to HA-trimer. The mAbs that did not bind to HA-trimer were mainly IgM+ in flow cytometry (62.2% of total non-binders) while the HA-trimer binding mAbs were mainly IgG+ (67.9% of total binders). Overall, the binding patterns of these mAbs, as tested by ELISA, corresponded to the binding patterns observed for the matching B cells in flow cytometry (**Figure 3A**). 70.6% of the HA-head B cells were also binding as mAb in ELISA to HA-head, 90% of the HA-stem B cells, and 73.3% of the HA-trimer-only B cells had a corresponding ELISA binding pattern. 23.5% of the HA-head B cells were not able to bind HA-head protein in ELISA but showed binding to the HA-trimer. Partly because of this, there were higher numbers of HA-trimer-only mAbs compared to the flow cytometry data (**Figure 3B**).

### Diverse VH-gene usage observed in HA-specific BCRs

Since the flow cytometry binding patterns were shown to correctly represent HA-trimer, head-, and stem-domain binding, a large-scale analysis of heavy chain BCRs of sorted HA-specific cells was performed for the selected six animals (n=536 sequences, post-infection). Subsequently, the same analysis was performed on the HA-specific mAbs that were described above.

First, the VH-gene distribution of the total IgM repertoire of each animal established through IgDiscover, without considering allelic variation, and this was compared with the VH-gene usage of the HA-specific B cell population (**Figure 3C**). The total IgM VH-gene repertoire used a wide range of VH-genes with natural variation in genes being expressed in the germline repertoire plus variation in expression level per animal. The HA-specific BCRs also used a highly diverse set of VH-genes and mostly mirrored the VH-gene usage in the naive IgM repertoire. However, some VH-genes that were infrequently used in the IgM repertoire were detected in relative higher proportions in the HA-specific repertoire, such as IGHV1-138. Furthermore, some VH-genes that were frequently used in the IgM repertoire were not present in the HA-specific BCRs, in particular IGHV3-NL_11 for A12 was highly represented in the IgM, but not found in the HA-repertoire. Hence, despite the broad VH-gene usage in HA-specific BCRs, there was some slight repertoire skewing, which was further apparent when the allelic variation within the VH-genes was considered (**Figure S8**).

On a more general level, the VH- and JH-family usage of HA-specific BCRs and the isolated HA-mAbs showed that genes of the IGHV4 and IGHJ4 family were used most often (**Figures 3B, D**, upper panels), in agreement with the frequency of gene usage observed in cynomolgus macaque total IgM repertoires (32). This gene preference was also seen in the HA-head, HA-trimer-only, and HA-head+stem BCRs but for HA-stem BCRs where both IGHV3 and IGHV4 (left panel) were equally dominant, and IGHJ5 was used more often than IGHJ4 (right panel). Similar IGHV-gene family distribution was observed in the HA-specific mAbs (lower panel), although here HA-stem mAbs showed relatively higher usage of IGHJ5 and IGHJ6 compared to the larger set of HA-stem specific BCRs. This suggests differences in VH and VJ repertoire usage for the HA-head and HA-stem specific B cell responses. Indeed, HA-head and HA-trimer-only BCRs used the combination IGHV4-IGHJ4 the most, while HA-stem BCRs preferred combination IGHV4-IGHJ5 (**Figure S9**).

### Stem-specific antibodies showed significantly less somatic hypermutations and longer CDRH3s compared to head-specific antibodies

Next, the IgM germline database was used to determine the SHM-level (at nucleotide level) for the HA-specific BCRs (**Figure 3E**). As expected, significantly fewer mutations were found in the BCRs from IgM+ HA-specific B cells compared to IgG+ HA-specific B cells. Interestingly, the HA-head specific BCRs had significantly higher SHM-levels compared to HA-stem specific BCRs (**Figure 3F**). The HA-trimer-only BCRs had similar low SHM-levels as the HA-stem BCRs. The difference in SHM-level between HA-head and HA-stem specific BCRs was also observed when analyzed for the IgG+ or IgM+ cells only (**Figure S10**). Hence, the difference in SHM between HA-head and HA-stem specific BCRs is not biased by the percentage of IgM+ cells. No correlation was found between the HA-specific SHM-level and the viral load, suggesting the level of virus replication and thus relative level of antigen exposure, does not directly translate into more affinity maturation of HA-specific BCRs (**Figure S11**).

Lastly, the CDRH3-length was evaluated of the post-infection HA-specific BCRs. It has been shown that the average CDRH3-length of cynomolgus macaques was 14.81 amino acids (aa) (32). In this study, the CDRH3-length of the HA-specific BCRs showed a bimodal distribution (**Figure 3G**), and with a median of 15 aa, a similar CDRH3-length compared to previous reports. There was no significant difference observed between IgM+ and IgG+ BCRs (**Figure S12**). When the HA-specific BCRs were subdivided into HA-head or HA-stem specific BCRs a significantly longer CDRH3 for the HA-stem specific BCRs (median 18 aa) compared to the HA-head specific BCRs (median 14 aa) was observed (**Figure 3H**), as also seen in the mAbs (**Figure S13**). These differences between HA-head and HA-stem specific BCRs could explain the bimodal pattern observed for the total HA-response. There was no significant difference in CDRH3 length in animals that had developed high levels of virus replication versus animals with low levels of replication (**Figure S14**).

## Discussion

While it has been assumed that the first exposure to influenza virus is shaping the immune response to future infections or vaccinations, the direct impact of primary exposure is difficult to study in humans because of their unknown and large infection and/or vaccination history (2). Therefore, in this study we describe an in-depth characterization, with the help of HA-trimer, HA-head, and HA-stem recombinant proteins (16), of the antibody responses after a primary H1N1_pdm2009_ influenza virus infection in cynomolgus macaques, a species that is phylogenetically closely related to humans and displays many immunological similarities (33, 34).

In humans it was observed that HA-head antibodies were dominant while stem antibodies were sparse (4, 8). However, induction of stem antibodies has been reported after vaccination or re-infection with hetero- or autologous influenza strains (8, 35–39). Also, animal studies showed that stem-directed antibodies can be elicited after infection/vaccination (7, 40), although other studies in ferrets and macaques reported a lack of stem responses after primary infection (41, 42). This different outcome may possibly be related by a difference in viruses used (H3N2) or HA-stem construct used for detection. The specificity of the HA-stem responses reported here was therefore confirmed further by competition ELISA using the C179 HA-stem mAb.

In this study, we observed early induction of HA-stem specific antibodies earlier after infection compared to HA-head antibodies. Early induction of HA-stem antibodies was also observed in several animal studies (4, 7). However, later time points were either not studied or a rapid decline of HA-stem antibodies was not observed (4). At the moment, there is no clear explanation for this discrepancy. The use of HA-head protein made the relative decrease of the HA-stem response even more striking. A note of caution is that since HA-head protein was expressed as a monomer and some HA-head binding antibodies might have been missed such as HA-head antibodies directed to the head interface or to the Ca-antigenic site which requires two monomers (43, 44), the total HA-head response might be underestimated. Nonetheless, the decrease in HA-stem responses is also obvious when related to the total HA-trimer response. As confirmed on B cell level, a significantly higher frequency HA-head specific B cells were found compared to HA-stem specific B cells at the latter timepoint. We hypothesize that HA-stem specific B cells and thereby HA-stem antibodies are part of the first antibody defense to the influenza virus infection, elicited by short lived plasma cells that have undergone limited affinity maturation. Indeed, we found that HA-stem specific antibodies displayed lower levels of SHM in their VH-genes compared to the HA-head specific antibodies. These data are consistent with the relative dominance of HA-head responses after infection or vaccination, and this may be due to the relative low exposure of the HA-stem domain.

We demonstrate that class-switching had occurred in the HA-specific B cells as these cells had significant higher levels of IgG+ compared to the total CD20+ B cell population. Importantly, while low levels of HA-specific B cells were detected in the two pre-infection samples, these had a naive B cell phenotype with low SHM-levels. After infection, no significant differences were observed in IgG+ expression between the different HA-binding domains, confirming that for all domains class switching had occurred at similar levels. This class switching can occur independent of the GC reaction (45), which could explain why despite similar levels of class switching the HA-head specific BCRs show increased levels of SHM compared to the stem-specific B cells.

In humans, long CDRH3 are shown to be associated with broadly reactive antibodies in influenza virus infections, as well as in HIV-1 and SARS-CoV-2 (29, 46–48). In HA-Abs, long CDRH3 regions were found in specific cases for both HA-head and HA-stem specific antibodies (10, 49–51), but a clear difference between HA-stem and HA-head CDRH3-length as shown here, has not been shown before. This observation might provide an indication of a selection towards longer CDRH3 for HA-stem antibodies and shorter CDRH3 for HA-head antibodies. Moreover, broadly reactive stem antibodies in humans are associated with VH1-69, a gene-family which is underrepresented in macaques (32) but the VH1 gene-family was present in the total HA-specific BCRs and previous reported HA-stem BCRs (52). We found, in contrast to humans, that IGHV3 and IGHV4 are highly represented in the HA-specific BCRs as reflected in the previous reported gene family usage (32, 52). In our study, allelic variation was observed in the IgM repertoire of the animals. The HA-specific BCR repertoire is limited to investigate whether these allelic variations affect the antibody response as previously reported (53).

By including both HA-head and HA-stem domain proteins we were able to identify a subset of B cells that bind to the tertiary HA-trimer structure, but neither to the trimeric stem nor monomeric head. This HA-trimer-only binding subset of B cells represents 50-80% of the HA-specific B cell population, similar to results obtained in humans (16). At present the function of the HA-trimer-only B cells is not yet clear, but they seem to deviate from standard HA-head or HA-stem binding specific B cells and more research is needed to understand what their function and possible role in protection is (16). Monoclonal antibodies (mAbs) were isolated and confirmed the binding patterns observed for the corresponding B cells in flow cytometry. Not all isolated mAb bound the HA-trimer, HA-head or HA-stem. This lack of binding may have been because all sequences were cloned into IgG vectors, while some B cells were originally IgM expressing, which could result in lower binding avidity when expressed as monovalent IgG compared to for example the original pentavalent IgM conformation.

To conclude, this study, for the first time, describes the primary response at the monoclonal antibody level after an influenza virus infection in cynomolgus macaques. We found an early induction of potentially broadly reactive stem antibodies followed by a subsequent focusing on the HA-head domain, involving higher levels of affinity maturation and memory B cell formation. Both stem- and head-specific responses display a highly diverse VH-usage with allelic variation within these genes. We propose that stem-specific antibodies provide first line of defense against a primary influenza virus infection and that these antibodies are produced by short lived plasma cells. In contrast, HA-head antibodies first undergo SHM in the germinal center resulting in high affinity antibodies that increase over time in the blood. Further research is needed to understand how a reinfection or vaccination influences the course of the specific HA-antibody responses. This knowledge is of importance for the development of future broadly protective vaccines.

## Data availability statement

The datasets presented in this study can be found in online repositories. The names of the repository/repositories and accession number(s) can be found below: https://www.ebi.ac.uk/ena/, ERR10322423-ERR10322438. The properties of heavychain BCRs can be found in **Supplemental Data Table 2**.

## Ethics statement

The animal study was reviewed and approved by Institutional Animal Care and Use committee of Biomedical Primate Research Centre (dierexperimentencommissie, DEC-BPRC; DEC#686).

## Author contributions

Conceptualization and study design: AA, PM, WB, GH, MG and GK. Methodology and analysis: AA, DM, SH, MH, DE, MCl, and MCo. AA, PM, MG, and GK wrote the first draft of the manuscript. All authors contributed to the article and approved the submitted version.

## Funding

This study is supported by internal funding of Biomedical Primate Research Centre. MG is supported by the AMC Fellowship from the Amsterdam UMC, Amsterdam, Netherlands.

## Conflict of interest

The authors declare that the research was conducted in the absence of any commercial or financial relationships that could be construed as a potential conflict of interest.

## Publisher’s note

All claims expressed in this article are solely those of the authors and do not necessarily represent those of their affiliated organizations, or those of the publisher, the editors and the reviewers. Any product that may be evaluated in this article, or claim that may be made by its manufacturer, is not guaranteed or endorsed by the publisher.

## Supplementary material

Supplementary Figure 1 Well characterized mAbs as protein-coating control. HA-head Ab 5J8, HA-stem Ab CR6261, and H3N2-HA-stem negative control Ab CR8020. Area under curve serial dilution (AUC), median and 95% CI of four different Ni-NTA plates, corresponding to the serological data.

Supplementary Figure 2 HA-stem competition ELISA with mAb C179 and two representative sera or vice versa. Sera was taken from day 0 (grey), day 14 (blue), and day 56 (yellow). Percentage of C179 was relative to the maximum binding of C179.

Supplementary Figure 3 Correlation plot of the viral load (AUC, from day 0 till 56) with HA-trimer (blue), HA-head (orange) and HA-stem (purple) antibody responses OD_450nm_ (AUC) at day 14 post-infection.

Supplementary Figure 4 Representative gating strategy to determine frequencies of HA-stem (sample 1 in BV421 label) and HA-head (sample 2 in BV421 label), and IgG and IgM frequencies.

Supplementary Figure 5 Correlation graph of HA-specific B cells (day 56) versus HA-specific serum antibodies (AUC) at day 14 post-infection. Spearman correlation, ns, not significant.

Supplementary Figure 6 Correlation graphs of HA-head (orange, upper panel) and HA-stem (purple, lower panel) B cell frequencies (day 56) versus HA-head B cells and HA-stem serum antibodies (AUC) at day 14 (left) and day 56 (right). Spearman correlation, ns, not significant.

Supplementary Figure 7 Correlation graphs of HA-head (orange, left) and HA-stem (purple, right) B cell frequencies (day 56) versus the viral load (AUC). Spearman correlation, ns, not significant.

Supplementary Figure 8 Dot plot of V-gene usage including allelic variation. Relative expression frequency per animal is depicted by the size of the dot, for both HA-specific BCR sequences and germline sequences.

Supplementary Figure 9 Bar chart representing the VH- and JH-gene family combinations of HA-specific BCRs, subdivided into the different binding domains obtained by flow cytometry. Percentages are relative to the number of sequences per binding domain group.

Supplementary Figure 10 SHM-level of VH-genes HA-head (orange) and HA-stem (purple), subdivided into IgG+ or IgM+. Statistical differences were calculated by using a Mann-Whitney test.

Supplementary Figure 11 Correlation graph of HA-trimer SHM-level and the viral load (AUC). Spearman correlation, median SHM-level with 95% CI, ns = not significant.

Supplementary Figure 12 CDRH3 density histogram of IgG (grey) and IgM (pink) HA-specific B cells. The dotted line is the median, both medians are at 15 amino acids. Statistical differences were determined with a Mann-Whitney test.

Supplementary Figure 13 CDRH3-length density histogram of HA-head (orange) and HA-stem (purple) isolated monoclonal antibodies. The dotted lines are the corresponding medians. Statistical differences were determined with a Mann-Whitney test.

Supplementary Figure 14 Density histogram showing the CDRH3-length of high viral load (green) and low viral load (red). The dotted line is the median, both medians are at 15 amino acids. Statistical differences were determined with a Mann-Whitney test.
