# Supplementary material for: Primary antibody response after influenza virus infection is first dominated by low-mutated HA-stem antibodies followed by higher-mutated HA-head antibodies
Source: Front Immunol. 2022 Nov 3;13:1026951. doi: 10.3389/fimmu.2022.1026951 (PMC9670313; doi:10.3389/fimmu.2022.1026951)
Supplement: Supplementary Figure 1 — Well characterized mAbs as protein-coating control. HA-head Ab 5J8, HA-stem Ab CR6261, and H3N2-HA-stem negative control Ab CR8020. Area under curve serial dilution (AUC), median and 95% CI of four different Ni-NTA plates, corresponding to the serological data. [file DataSheet_1.pdf]

Supplemental figures, corresponding to manuscript titled:

"Primary antibody response after influenza virus infection is first dominated by low-mutated HA-stem antibodies followed by higher-mutated HA-head antibodies"

Figure S1:

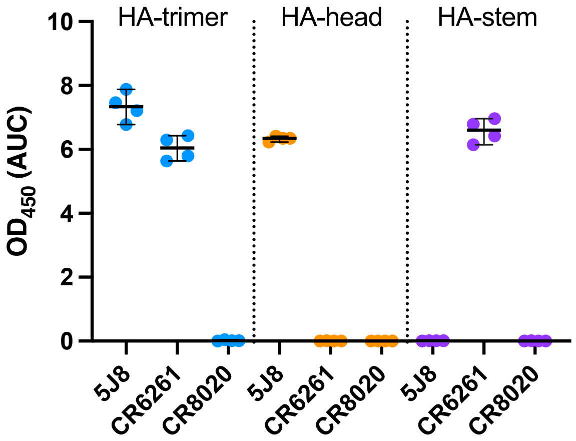

Figure S2:

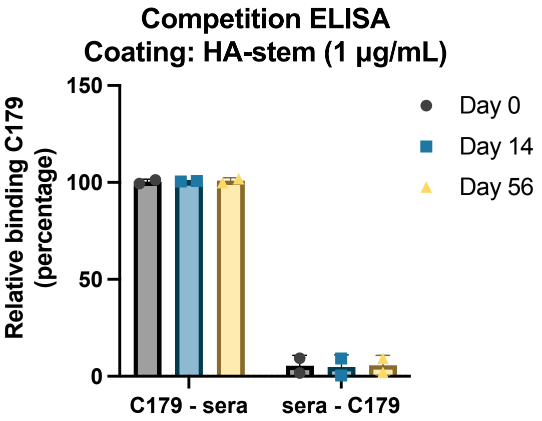

Figure S3:

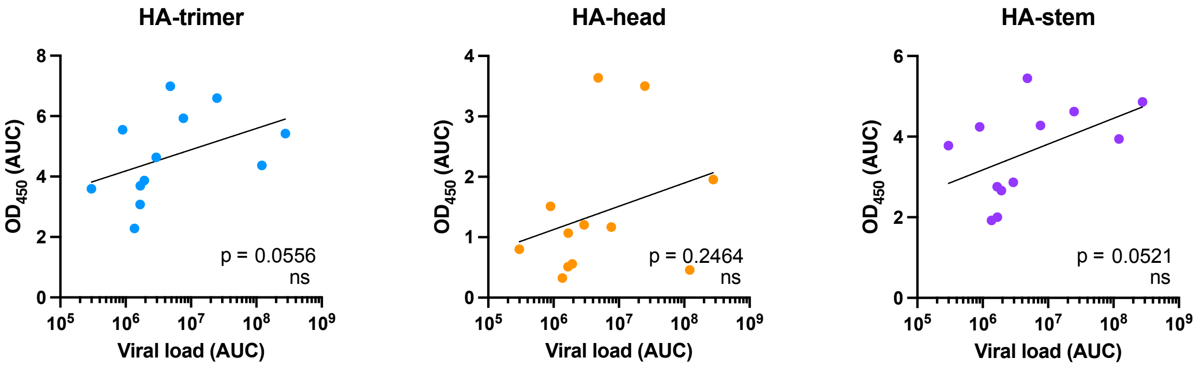

Figure S4:

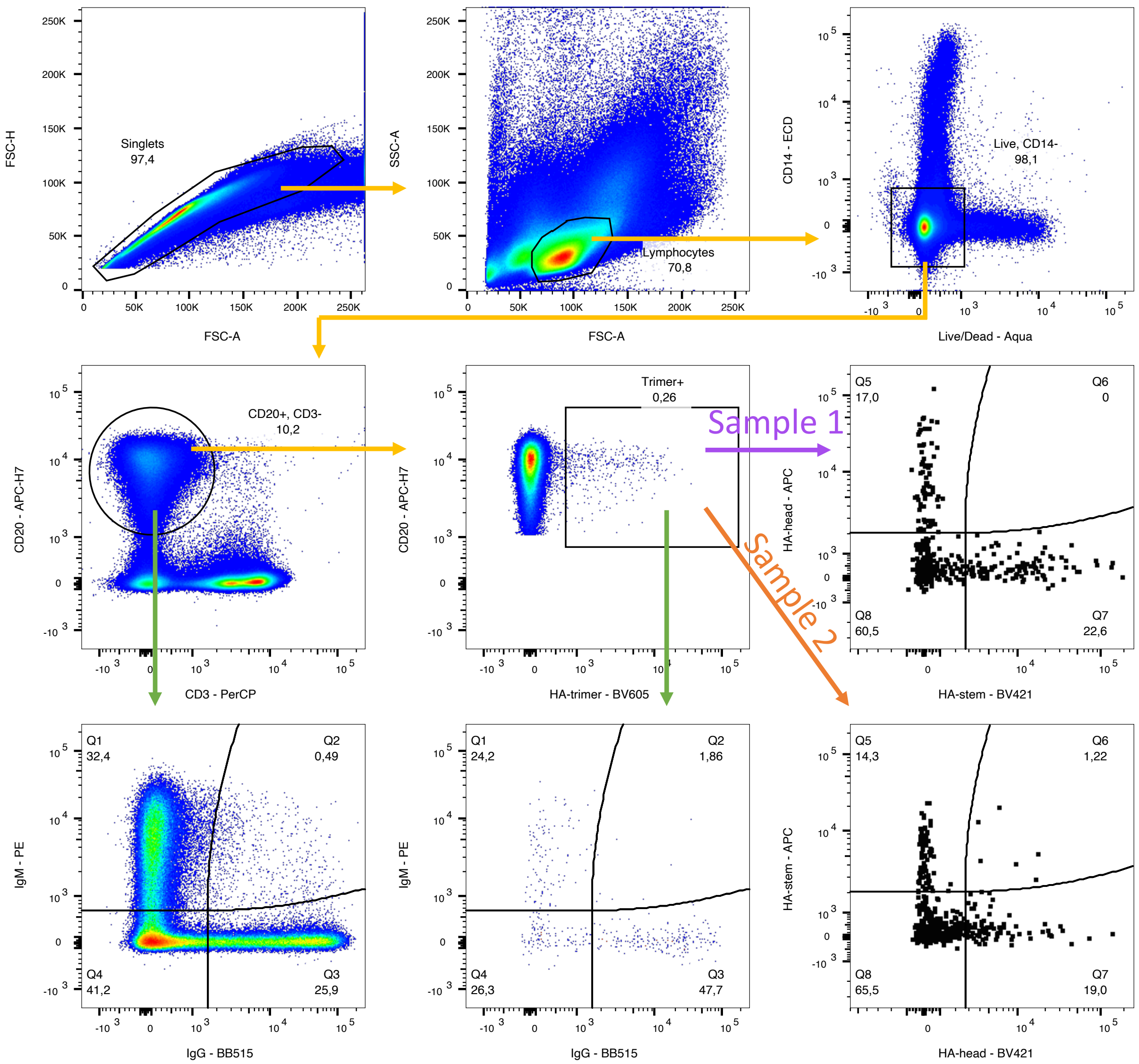

Figure S5:

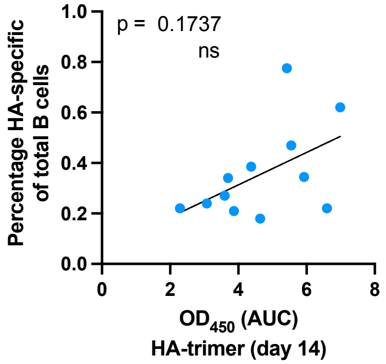

Figure S6:

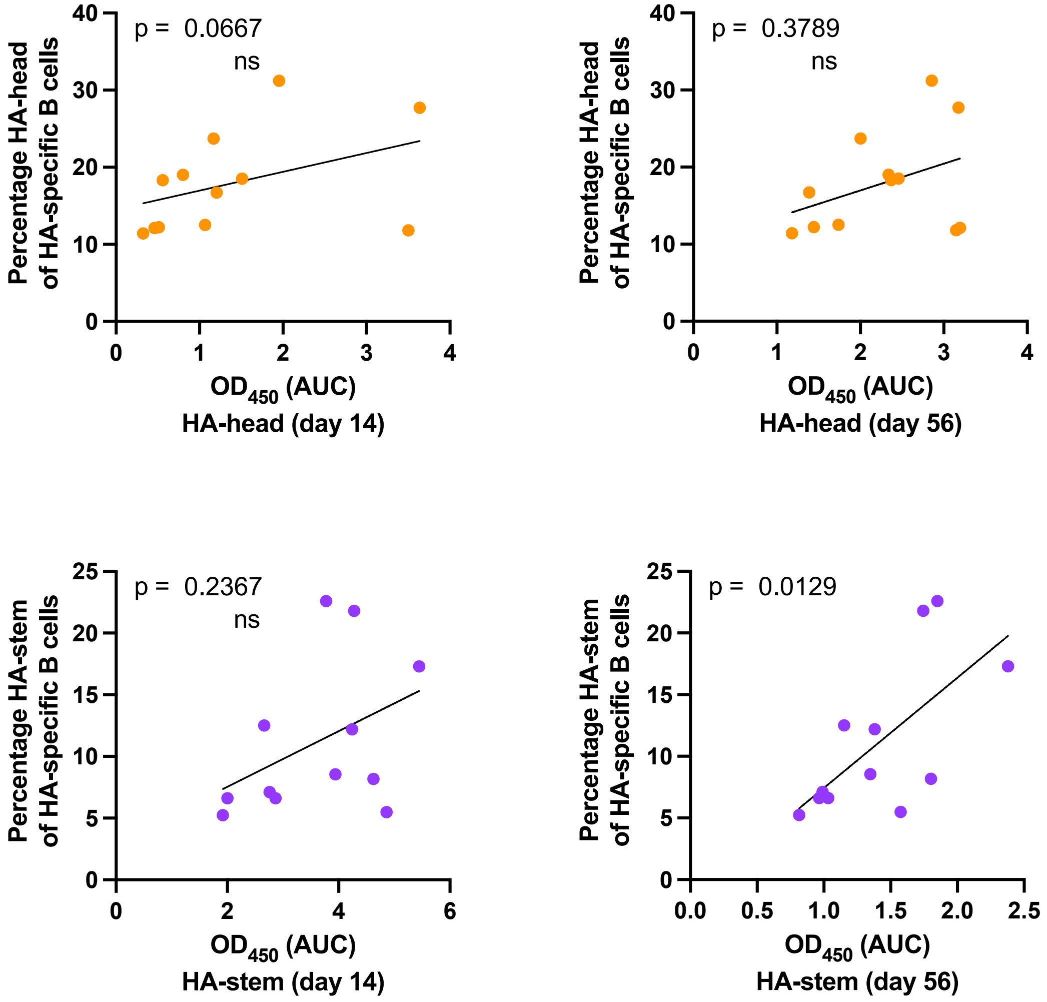

Figure S7:

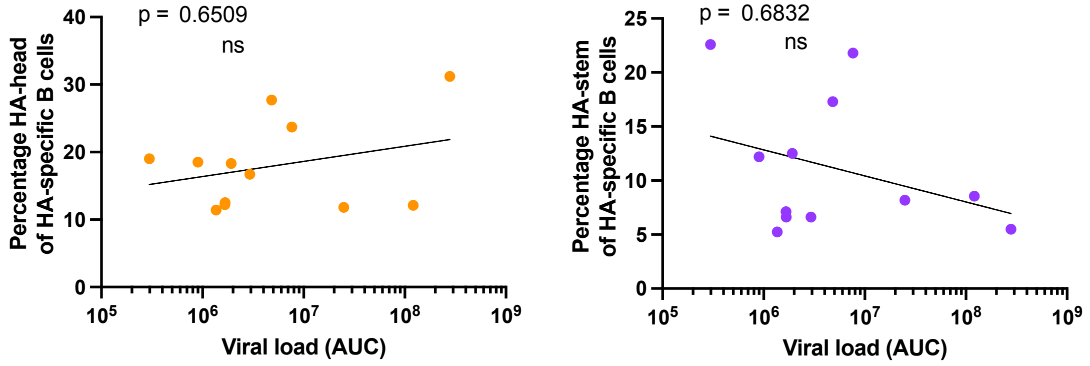

Figure S8:

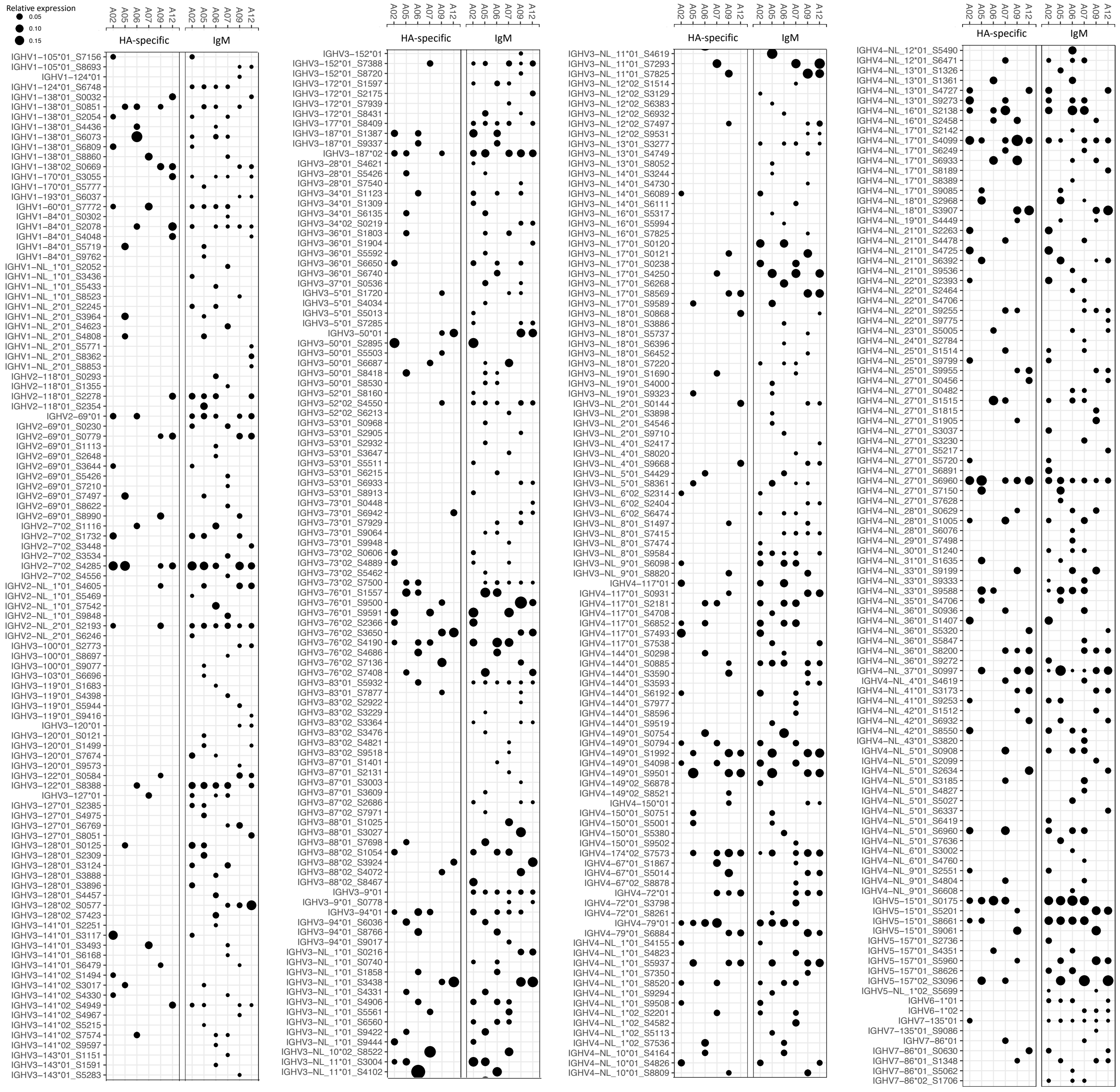
Relative expression
0.05
0.10
0.15
A02
A05
A06
A07
A09
A12
HA-specific
IgM

Figure S9:

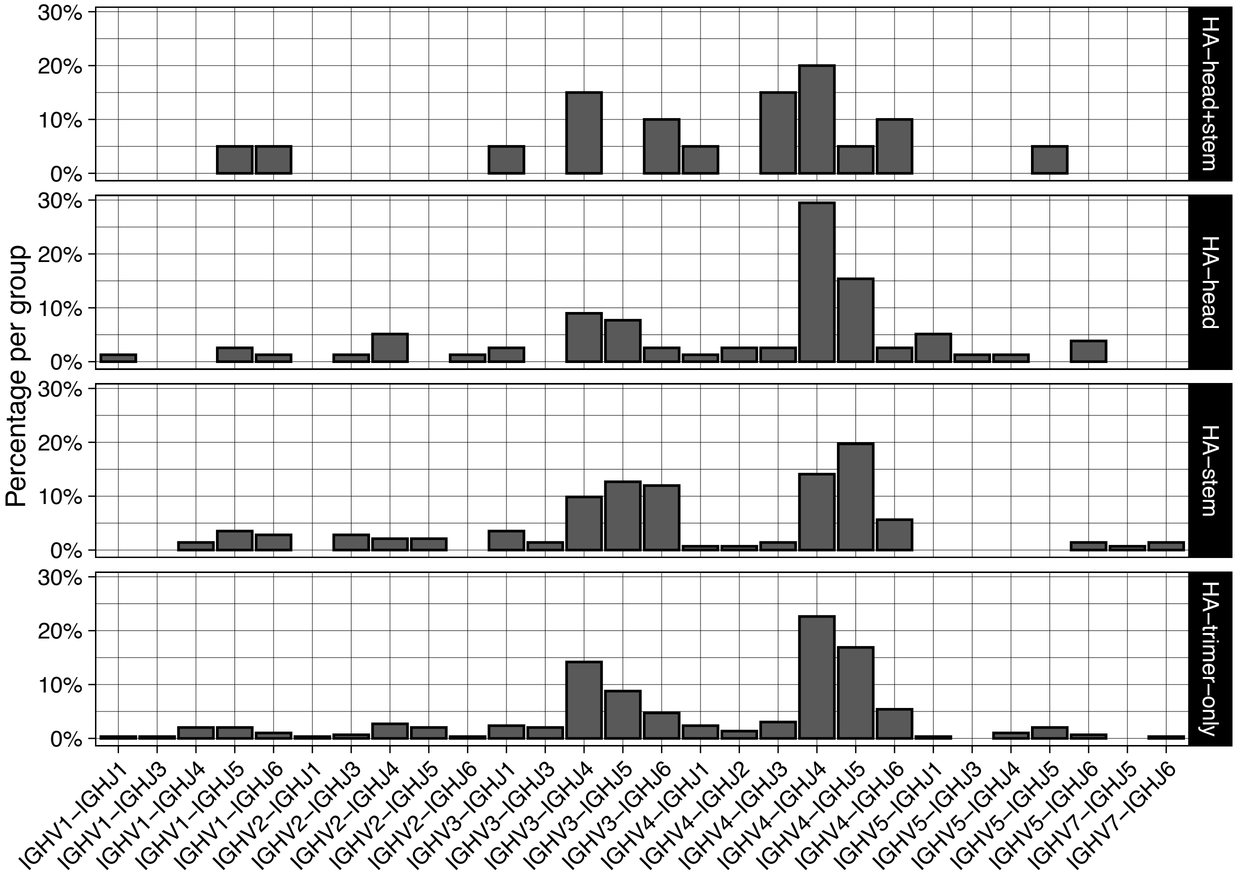

Figure S10:

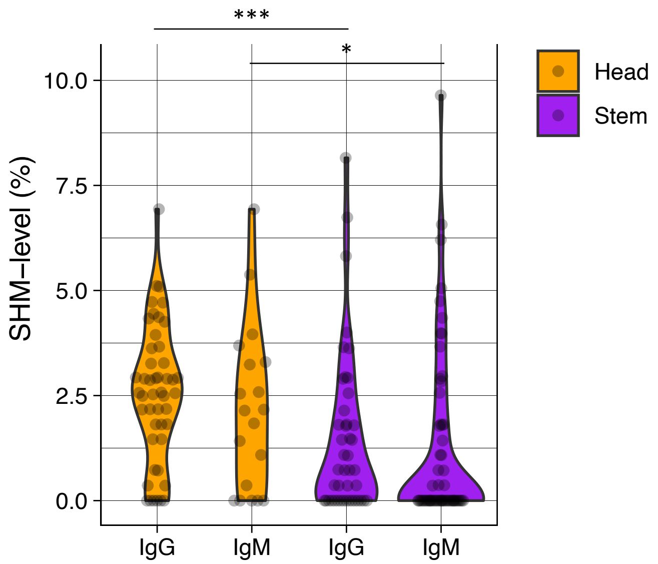

Figure S11:

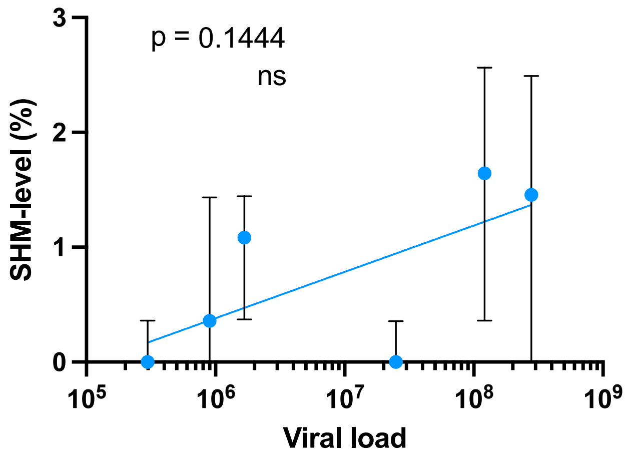

Figure S12:

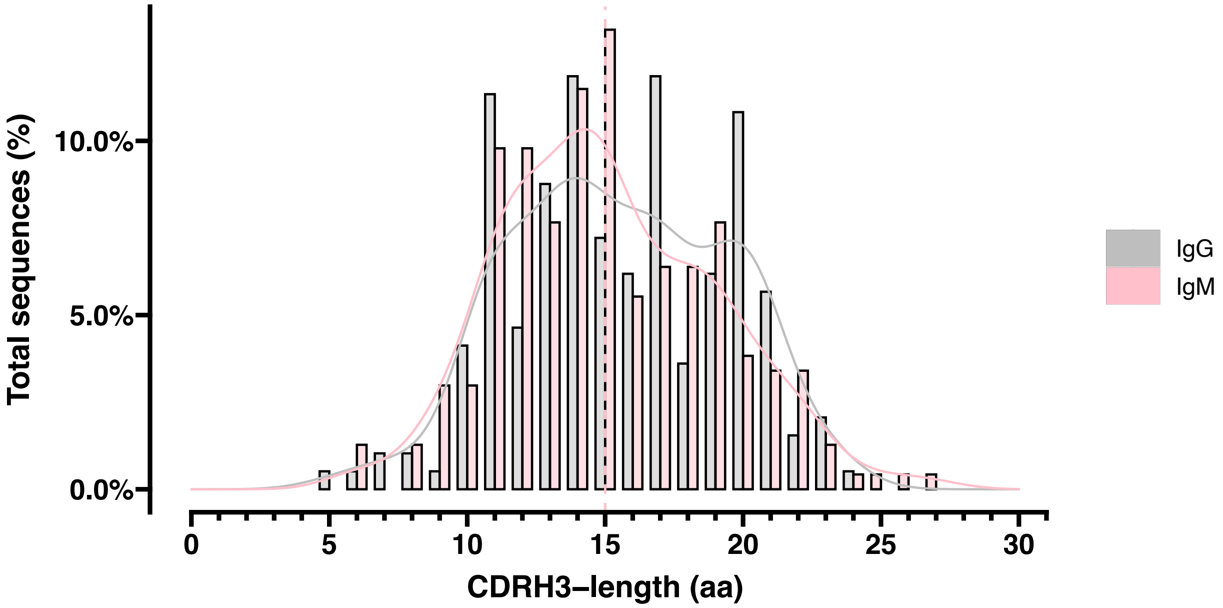

Figure S13:

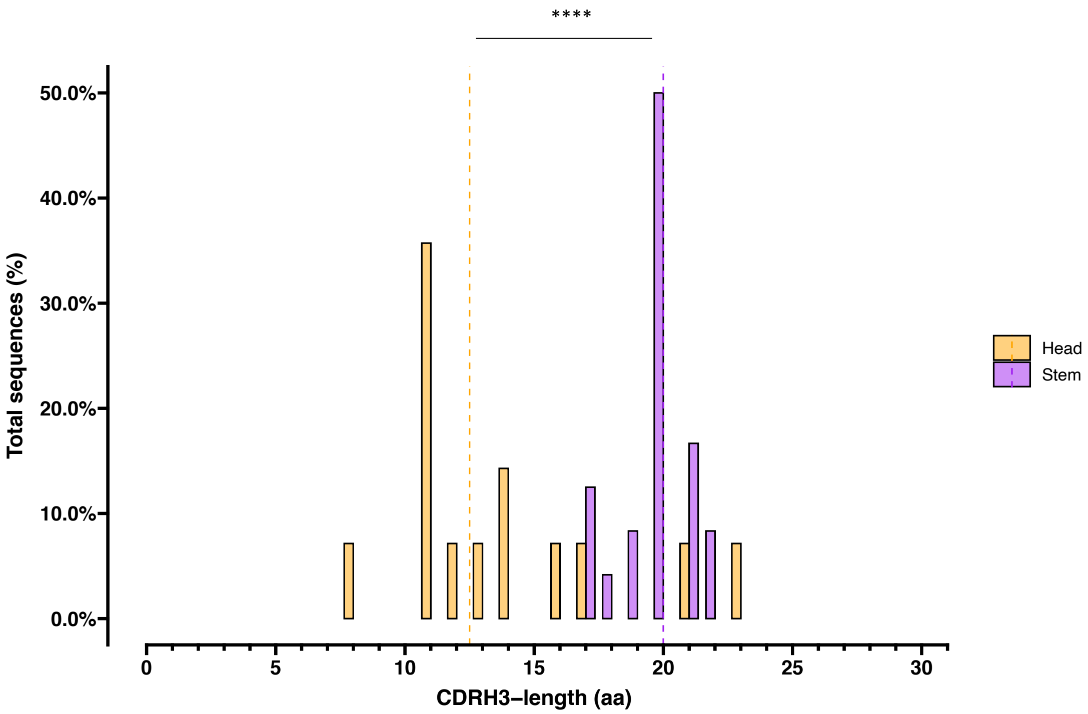

Figure S14:

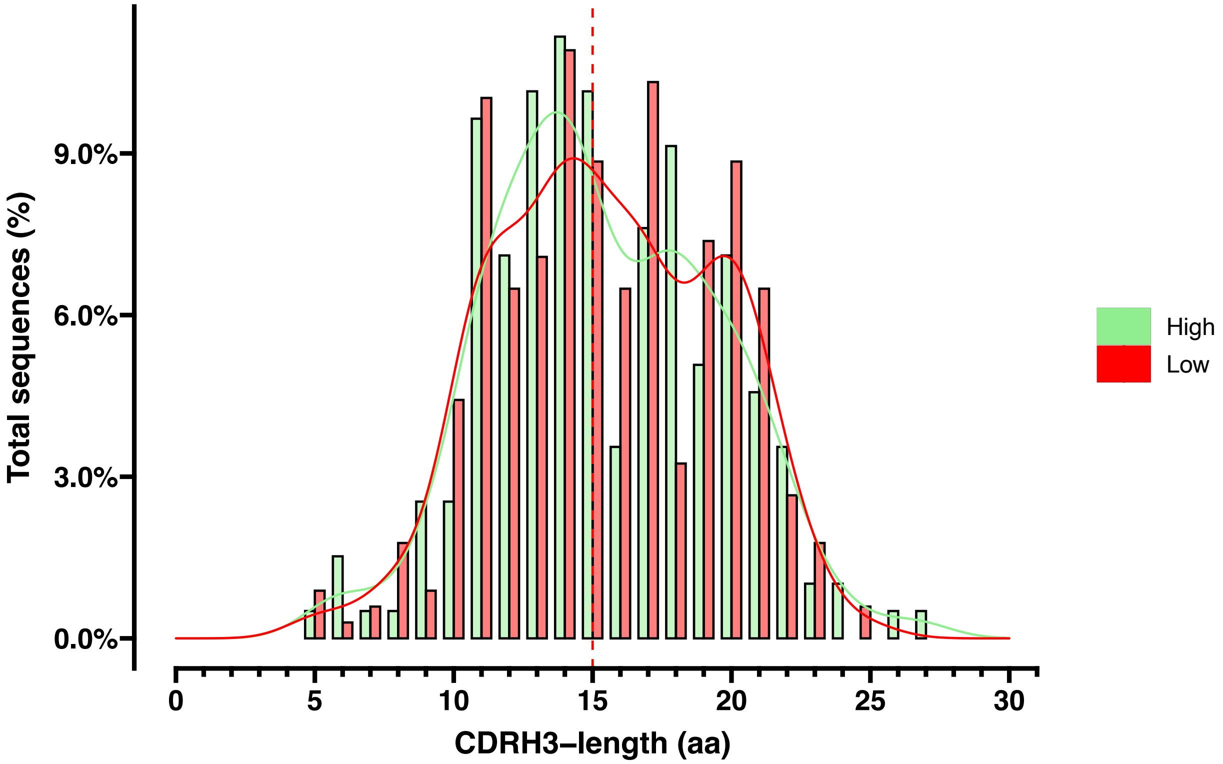

Table S1: six animals selected for HA-specific BCR analysis

Name	Viral load (AUC)	Dose
**A02**	1.67×10^{6} (Low)	Low
**A05**	2.50×10^{7} (High)	Low
**A06**	2.98×10^{5} (Low)	Low
**A07**	2.79×10^{8} (High)	High
**A09**	8.98×10^{5} (Low)	High
**A12**	1.21×10^{8} (High)	High
